# Dietary Patterns and Maternal Anthropometry in HIV-Infected, Pregnant Malawian Women

**DOI:** 10.3390/nu7010584

**Published:** 2015-01-14

**Authors:** Roshan T. Ramlal, Martin Tembo, Caroline C. King, Sascha Ellington, Alice Soko, Maggie Chigwenembe, Charles Chasela, Denise J. Jamieson, Charles van der Horst, Margaret Bentley, Linda Adair, the BAN Study Team

**Affiliations:** 1 University of North Carolina, Chapel Hill, NC 27599, USA; E-Mails: charles_vanderhorst@med.unc.edu (C.H.); pbentley@unc.edu (M.B.); linda_adair@unc.edu (L.A.); 2 UNC Project Malawi, Tidziwe Center, Mzimba Road, Lilongwe, Malawi; E-Mails: martintembo@gmail.com (M.T.); asoko@unclilongwe.org (A.S.); mchigwenembe@unclilongwe.org (M.C.); Charles.Chasela@wits.ac.za (C.C.); 3 U.S. Centers for Disease Control and Prevention, 4770 Buford Highway, Atlanta, GA 30341, USA; E-Mails: ccking@cdc.gov (C.C.K.); sellington@cdc.gov (S.E.); djamieson@cdc.gov (D.J.J.); 4 Division of Epidemiology and Biostatistics, Faculty of Health Sciences, University of Witwatersrand, Johannesburg, 2193, South Africa

**Keywords:** maternal diet, nutrition, pregnancy, HIV, anthropometry, Malawi, cluster analysis

## Abstract

Diet is a modifiable factor that can contribute to the health of pregnant women. In a sample of 577 HIV-positive pregnant women who completed baseline interviews for the Breastfeeding, Antiretrovirals, and Nutrition Study in Lilongwe, Malawi, cluster analysis was used to derive dietary patterns. Multiple regression analysis was used to identify associations between the dietary patterns and mid-upper arm circumference (MUAC), arm muscle area (AMA), arm fat area (AFA), and hemoglobin at baseline. Three key dietary patterns were identified: animal-based, plant-based, and grain-based. Women with relatively greater wealth were more likely to consume the animal-based diet, which had the highest intake of energy, protein, and fat and was associated with higher hemoglobin levels compared to the other diets. Women with the lowest wealth were more likely to consume the grain-based diet with the lowest intake of energy, protein, fat, and iron and were more likely to have lower AFA than women on the animal-based and plant-based diets, but higher AMA compared to women on the animal-based diet. Pregnant, HIV-infected women in Malawi could benefit from nutritional support to ensure greater nutrient diversity during pregnancy, when women face increased nutrient demands to support fetal growth and development.

## 1. Introduction

Infection with human immunodeficiency virus (HIV) increases resting energy expenditure and may limit dietary intake and reduce nutrient absorption [1]. In addition, nutritional status can influence the progression of HIV disease. Women are disproportionately affected by HIV compared to men. Across all age groups there is a higher rate of HIV prevalence among women than among men. Approximately 60% of adults living with HIV in Malawi are female [2]. HIV-infected pregnant women are particularly at risk since they have additional nutrient demands to support fetal growth and development [3]. Diet during pregnancy is a potentially modifiable factor that can contribute to the health of pregnant women and improve birth outcomes [4].

Women account for an estimated 60% of HIV infections in sub-Saharan Africa, where food availability, malnutrition, and infectious disease morbidity can vary substantially by season due to cycles of rainfall and agricultural production [5,6]. In Malawi, the annual famine season extends from August to March. The typical Malawian diet includes a staple food, such as *Nsima*, a thick maize porridge molded into patties, served with beans, vegetables, or relish for flavor. Most animal-source foods, rich in protein and micronutrients, are expensive and scarce. The current study used cluster analysis to identify three dietary patterns among 577 HIV-positive pregnant women in Lilongwe, Malawi, and compare sociodemographic and anthropometric characteristics of the women across diet patterns.

## 2. Experimental Section

Dietary recalls were collected from women who enrolled in the Breastfeeding, Antiretrovirals, and Nutrition (BAN) Study from April 2004 to March 2006. The BAN Study recruited women from four antenatal clinics with outreach to all pregnant women in Lilongwe [7]. At the first antenatal visit, consenting women were screened for pre-delivery study inclusion criteria: (1) ≤30 weeks gestation based on last menstrual period or fundal height; (2) ≥14 years of age; (3) confirmed HIV infection; (4) hemoglobin ≥ 7 g/dL; (5) CD4 count ≥ 200 cells/µL; (6) no prior antiretroviral medication use; (7) normal liver function tests (<2.5 times the upper limit of normal); (8) no serious complications of pregnancy; and (9) not previously enrolled in the BAN Study. At the second antenatal visit (referred to as the baseline visit), women completed a standardized interview, physical exam, and specimen collection. All women received prenatal iron folate tablets (200 mg ferrous sulfate and 0.25 mg folic acid) per Malawi standard of care and all women diagnosed with malaria were treated. The BAN Study included up to 5 antenatal visits, a labor and delivery visit, and 14 postpartum visits, with randomization occurring within 1 week of delivery to a 2-arm nutritional intervention to prevent maternal depletion and a 3-arm antiretroviral intervention to prevent HIV transmission during breastfeeding [8,9]. The BAN Study protocol was approved by the Malawi National Health Sciences Research Committee and the institutional review boards at the University of North Carolina at Chapel Hill and the U.S. Centers for Disease Control and Prevention (ClinicalTrials.gov identifier NCT00164762).

From April 2004 to March 2006, 738 women completed baseline interviews and delivered live singletons. Trained BAN Study staff collected 24-h dietary recalls at the baseline visit. Recipes and ingredients of mixed dishes were recorded. Women were asked if the diet recall indicated “typical intake” and were prompted for an explanation if it did not. Food models and utensils were used to help respondents recall portion sizes and proportion of mixed dishes consumed. A Malawi nutrient food composition table (FCT) [10] was used to estimate nutrient intakes. Supplemental nutrient information was obtained from Malawi’s Ministry of Health’s FCT, a Tanzanian FCT [11], and the USDA nutrient database [12]. The resulting database had nutrient content of individual food items as well as mixed dishes based on standard recipes. Dietary recalls were reviewed for plausible portion sizes and nutrient intakes; 577 recalls were deemed reliable for this analysis. Independent sample t-tests were used to evaluate potential selection bias.

Weight, height, mid-upper arm circumference (MUAC), and triceps skinfold thickness were measured by trained BAN Study staff. Weight was measured to the nearest 100 g at each visit with an electronic scale checked regularly with a standard 5 Kg weight. Height was measured with a wall-mounted stadiometer. At each visit, MUAC was measured at the midpoint between the olecranon and acromion process, to the nearest 0.1 cm using an insertion tape, while the arm hung freely at the side. Triceps skinfold thickness was measured in triplicate at each visit using Lange Calipers. The mean of the three measurements was used with MUAC to derive arm muscle area (AMA = [MUAC − (triceps skinfold × π)]^2^/4π) and arm fat area (AFA = MUAC^2^/4π − AMA) [29]. Only the initial baseline measure, which was typically in the second trimester, was used in this analysis. Infant weights were measured at delivery or at the first visit post-delivery for home deliveries.

Anemia was defined as mild (<12 g/dL) or moderate (<10 g/dL) in accordance with the 2004 Malawi Demographic Health Survey (MDHS) definitions [13].

### Statistical Analysis

To identify dietary patterns, we categorized food items into 12 groups: (1) grains; (2) legumes, groundnuts, seeds and soy; (3) tubers; (4) fruit; (5) leafy green vegetables; (6) fish; (7) meat poultry, and eggs; (8) dairy; (9) fats and oils; (10) sugars, candy, soft drinks; (11) hot beverages; and (12) miscellaneous. Nutrient densities (grams/total calories) of each food group were calculated and daily intake was used in the cluster analysis. Standardization by energy contribution helps to remove dietary variations due to differences in age, body size, and physical activity and to retain the proportional differences in food intake patterns [14,15]. Prior to cluster analysis, values were transformed into sample-specific Z-scores so that food intake differences between clusters could be illustrated and compared. Dietary patterns were generated by K-means cluster analysis [16] based on nutrient densities of each food group. We examined solutions with 2 to 5 clusters to evaluate which set of clusters was more meaningful to define dietary patterns.

The STATA [16] kmeans cluster method was used to group women according to nutrient densities of intake derived from each of the 12 food groups. The three-cluster solution was determined most appropriate based on power requirements and sufficient representation of typical dietary patterns of Malawian women with nutritionally meaningful variation between clusters. Sociodemographic characteristics, anthropometrics, and macro-nutrient intakes were compared across the three clusters using chi-square tests for categorical variables and a one-factor analysis of variance (ANOVA) for continuous variables. If the global p-value was significant (<0.05), pairwise comparisons were made using a Bonferroni correction for multiple comparisons (*p*-value considered significant if <0.017). Multivariable linear regression was used to examine associations between dietary patterns and maternal nutritional indicators: MUAC, AMA, AFA, and hemoglobin. The multivariable models included a variable indicating whether or not the 24-h dietary recall reflected the participant’s typical diet. Exposure to the famine season, defined by the number of days during the previous month that were spent in the famine season (August-May), was also included as a covariate. A wealth index of five quintiles was derived using principal component analysis of household characteristics: house construction (type of walls, floors, and roof), number of rooms and residents, electricity, refrigeration, sanitation, water source and cooking fuel source [17]. Other covariates included in the regression models were total energy intake, age, parity, education, maternal employment status, height and CD4 count. Multivariable models were assessed for statistically significant interactions (*p* < 0.20) between the dietary patterns and exposure to the famine season and between dietary patterns and wealth status [18].

## 3. Results

There were no significant differences (*p* < 0.05) in anthropometric, clinical, or seasonal indicators between the 577 women included (Table 1) and those excluded from this study, suggesting no selection bias. The mean daily energy intake was low (1378 kcal, interquartile range: 778, 1813), and over half of the women had mild (32.1%) or moderate (23.7%) anemia. No significant interactions were detected between dietary patterns and exposure to famine season and between dietary patterns and wealth status.

**Figure 1:**
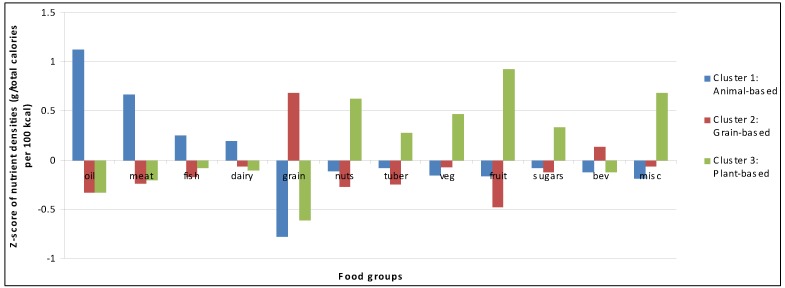
Cluster analysis of dietary patterns among HIV-infected Malawian pregnant women.

The three diet pattern clusters were labeled: 1) animal-based; 2) grain-based; and 3) plant-based. By definition, Cluster 1 had the highest intake of fish, meat, poultry, fat/oil, eggs and dairy, providing diets rich in energy and micronutrients (Figure 1). A typical meal in this cluster was a meat stew or soup with added oil or dried fish. Cluster 2 represents a grain-based diet of maize, rice, and millet, providing low levels of energy and micronutrients. A typical meal in this cluster was a plate of nsima only. Cluster 3 represents a mostly plant-based diet of leafy vegetables, beans, legumes, tubers, nuts, and fruits providing high levels of protein-rich or micronutrient-rich carbohydrates. A typical meal in this cluster was nsima with mustard greens and groundnut flour.

**Table 1:** Baseline demographics, nutritional status, caloric intake, and clinical characteristics among 577 pregnant women participating in the BAN Study.

Characteristic	*N* = 577
Age (year) [mean ± SD] ^1^	25.9 ± 4.9
*Education*	
No school (%)	11.3
Primary (%)	52.8
Secondary or higher (%)	35.9
*Occupation Status*	
Unemployed (%)	81.3
Employed (%)	18.7
*Experienced famine season*	
None (%)	39.7
Some (%)	14.6
All (%)	45.7
Parity (live births) [mean ± SD]	1.7 ± 1.3
Gestational age (weeks) [mean ± SD]	25.2 ± 5.4
CD4 count (cells/uL) [range (IQR) ^2^]	442 (325–601)
Hemoglobin (g/dL) [mean ± SD]	10.8 ± 1.2
Daily energy total intake (kcal) [mean ± SD]	1378 ± 821
Mid-upper arm circumference (cm) [mean ± SD]	26.4 ± 2.6
Arm muscle area (cm^2^) [mean ± SD]	36.6 ± 6.4
Arm fat area (cm^2^) [mean ± SD]	19.5 ± 7.8

^1^ SD: standard deviation; ^2^ IQR: interquartile range.

Comparisons across the three clusters indicated that employment status and median CD4 count did not differ significantly but mean age (*p* = 0.02) and education (*p* = 0.05) did. However, in pairwise comparisons with Bonferroni correction, age and education were not significant. The clusters differed by wealth and exposure to the famine season. Significantly more women in the grain-based cluster were in the lowest wealth index quintile compared to women in the animal-based cluster, and more were exposed to the famine season compared to either the animal-based or plant-based clusters (Table 2). Women in the grain-based cluster compared to the animal-based cluster also had a history of more live births. Women in the grain-based diet cluster consumed significantly fewer calories, protein, fat, and iron than women in the animal-based or plant-based diets (Table 2). They also had significantly lower carbohydrate intake than women in the plant-based cluster. In univariate analysis, women in the grain-based cluster had significantly lower AFA compared to women in the plant-based cluster (Table 2). However, in multivariable analysis, the predicted mean difference in AFA was significant comparing the grain-based cluster to both the plant-based (−2.47 cm^2^ lower) and animal-based (−2.09 cm^2^ lower) clusters (Table 3). Compared to women in the animal-based cluster, women in the grain-based cluster had significantly higher AMA and lower hemoglobin level in both univariate and multivariable analysis. The predicted mean increase in AMA was 1.86 cm^2^ and the predicted decrease in hemoglobin level was −0.27 g/dL. The animal-based diet cluster had the highest intake of energy, protein, and fat at levels significantly above those of the plant-based cluster. In contrast, the plant-based diet had the highest intake of carbohydrates at a level significantly above that of the animal-based diet. While there were no differences between in maternal anthropometrics of women in the animal-based and plant-based diet clusters in univariate analysis, in multivariable analysis, the plant-based cluster had a significantly lower predicted mean difference in hemoglobin level (0.32 g/dL) than the animal-based cluster (Table 3). These measures did not have any clinical significance; however, it describes the association between prenatal body composition and dietary profiles. There were no differences in mean MUAC between the clusters in unadjusted (Table 2) or adjusted analysis. Furthermore, there were no differences between the diet clusters in infant weight at delivery.

**Table 2:** Demographic, nutrient, and clinical indicators by the 3 diet clusters among 577 pregnant women participating in the BAN Study.

Indicators	Animal-Based ^1^ (*n* = 160)	Grain-based ^2,3^ (*n* = 254)	Plant-based ^4^ (*n* = 163)	*P* value for Pairwise Cluster Comparisons ^5^		
				Animal *vs.* Grain	Animal *vs.* Plant	Grain *vs.* Plant
*Characteristics* ^6^						
Age (year) (mean ± SD)	25.0 ± 4.4	26.2 ± 5.1	26.2 ± 4.9	-	-	-
No school (*n* = 65) [*n*(%)]	11 (6.9)	37 (14.5)	17 (10.4)	-	-	-
Lowest wealth index (*n* = 108) [*n*(%)]	17 (15.7)	63 (58.3)	28 (25.9)	0.001	-	-
Parity > 2 (*n* = 133) [*n*(%)]	23(14.3)	67 (26.4)	43 (26.3)	0.014	-	-
Any famine season exposure (*n* = 348) [*n*(%)]	83 (51.9)	180 (70.9)	85 (51.2)	<0.001	-	<0.001
*Nutrition status*						
Daily energy total intake (kcal)	1776.8 ± 859.5	1083.5 ± 672.2	1445.4 ± 818.5	<0.001	<0.001	<0.001
Carbohydrates (g/day)	195.8 ± 116.3	201.1 ± 128.9	237.4 ± 135.3	-	0.010	0.014
Protein (g/day)	69.3 ± 57.1	32.6 ± 28.3	47.1 ± 35.9	<0.001	<0.001	0.001
Total fat (g/day)	82.9 ± 51.2	19.9 ± 18.5	41.2 ± 43.8	<0.001	<0.001	<0.001
Iron (g/day)	10.1 ± 9.9	6.9 ± 5.9	11.3 ± 8.4	<0.001	-	<0.001
Hemoglobin (g/dL)	11.0 ± 1.1	10.7 ± 1.2	10.7 ± 1.1	0.017	-	-
MUAC (cm)	26.3 ± 2.7	26.3 ± 2.6	26.8 ± 2.6	-	-	-
AMA (cm^2^)	35.3 ± 6.2	37.3 ± 6.5	37.0 ± 6.3	0.007	-	-
AFA (cm^2^)	20.2 ± 8.0	18.7 ± 7.0	20.7 ± 8.5	-	-	0.003
Infant weight at delivery (g)	3058.6 ± 431.4	2976.9 ± 434.4	3053.9 ± 395.2	-	-	-

^1^ Food pattern of high fish, meat and oil; ^2^ Food pattern of high grain and grain-derived foods; ^3^ Food or food group contributed the relatively lowest mean intake across the 3 clusters; ^4^ Food pattern of high leafy vegetables, nuts, tubers, fruits; ^5^ Determined by *t* tests or chi-square tests (values *p* < 0.017, Bonferroni adjustment for multiple comparisons); ^6^ CD4 and maternal work status are not included in the table because no significant differences were found when comparing across three clusters (*i.e.*, global *p*-value > 0.05).

**Table 3:** Predicted mean difference in arm muscle area (AMA), arm fat area (AFA), and hemoglobin level (Hb) of women in the three clusters of dietary patterns.

Outcome	Mean Difference	95% CI		*p* value
*AMA* ^1^ *(cm^2^)*				
Grain (compared to animal)	1.86	0.53	3.19	0.01
Plant (compared to animal)	1.23	−0.14	2.60	0.08
Plant (compared to grain)	−0.63	−1.89	0.63	0.33
*AFA* ^1^ *(cm^2^)*				
Grain (compared to animal)	−2.09	−3.75	−0.44	0.01
Plant (compared to animal)	0.38	−1.33	2.08	0.67
Plant (compared to grain)	2.47	0.90	4.03	<0.01
*Hb* ^1^ *(g/dL)*				
Grain (compared to animal)	−0.27	−0.52	−0.01	0.04
Plant (compared to animal)	−0.32	−0.59	−0.07	0.01
Plant (compared to grain)	−0.06	−0.30	0.18	0.62

^1^ Adjusted for total energy intake, season, age, parity, education, wealth index, maternal employment status, height and CD4 count.

## 4. Discussion

To our knowledge, this is the first study to use cluster analysis to examine dietary patterns among pregnant, HIV-infected women in sub-Saharan Africa. This sample represents a young group of HIV-positive Malawian women with low levels of education, high unemployment, low parity and relatively high CD4 counts, indicative of good immune status. The women in our study had relatively healthy values of MUAC, AMA, and AFA that are representative of Malawian women [19,20,21]. Despite the provision of prenatal iron tablets, women in the study were mildly anemic. However, anemia is common among HIV-infected women in resource-limited areas where nutritional deficiencies are compounded by parasitic infections, compromised immunity, and the hematological consequences of chronic and systemic inflammation [22,23].

Three key dietary patterns were identified and were associated with differences in nutrient quality, sociodemographic characteristics, and nutritional outcomes for the women. Women in the lowest wealth index were more likely to consume grain-based diets with the lowest intake of energy, protein, fat, and iron. A higher proportion of women on grain-based diets were exposed to the famine season, which would be expected to negatively impact nutritional outcomes, and had lower AFA compared to women on the plant-based and animal-based diets [24]. However, women on grain-based diet had a higher AMA compared to women on animal-based diets. The higher lean muscle mass among women consuming grain-based diets may reflect increased manual labor, such as farming. Women on animal-based diets had the highest intake of energy, protein, and fat and had significantly higher mean difference in hemoglobin level compared to women on the plant-based and grain-based diets. In addition, high intake of phytate and low intake of heme-iron results in lower bioavailability of iron, which would further compound low iron intake.

While multiple 24-h recalls would be ideal for analyzing associations between diet and maternal body composition [25], only one 24-h recall was collected in the BAN Study. Although one study suggested that Malawian diets among low-income women have little daily variation [10], another recent study among pregnant, rural Malawian women found high within and between individual variance in energy intakes [19]. As such, our use of only one dietary recall per participant may result in some diet misclassification. Further misclassification may arise due to the use of nutrient content based on the raw ingredients of each dish although several dishes were consumed cooked.

With more than 500 dietary recalls from HIV-infected, pregnant Malawian women, we derived dietary patterns typical of Malawians using cluster analysis and showed that the patterns differed markedly in diet quality. The use of dietary patterns rather than individual nutrients in assessing diet and health relationships has emerged as a method of capturing the total diet [26,27,28,29]. This type of evaluation allows for the examination of associations of multiple dietary components with the outcome of interest [15]. Analyzing foods instead of nutrients makes it easier to translate results into intervention messages. Furthermore, seasonal and cultural factors influence diet patterns, which in turn affect nutrient intakes.

Some limitations of the cluster analysis method include inherent subjectivity, which occurs throughout the pattern analysis because investigators must decide how to collapse the data into food groups and how to quantify the contribution of each food group to the total diet [26,30,31]. Additionally, the label attributed to a given diet pattern is a crude and sometimes subjective description, based either on a quantitative measure of the predominant food group consumed or a qualitative measure of health of the diet. To the best of our ability, we objectively used food composition databases and substantial knowledge of Malawian diets to select food groups and clusters most representative of diet patterns of Malawian women.

Although WHO and Malawi guidelines for ART initiation has changed and many of the subjects in this study would be eligible for ART, pregnant, HIV-infected women in Malawi could still benefit from nutritional counseling and food supplementation to ensure greater nutrient diversity during the gestational period, when they face additional nutrient demands to support fetal growth and development.

## 5. Conclusions

In summary, we identified three patterns of diet among pregnant HIV-infected women in Malawi, and these patterns were related to socioeconomic status as well as nutritional outcomes. A primarily grain-based diet had the least nutrient quality, and women consuming this diet appeared to be the most vulnerable to adverse nutritional outcomes. This study describe typical diets of HIV-infected, pregnant women and highlights poor quality of maternal diets that need to enhanced to meet demands of this particular group of pregnant women, vulnerable to both HIV and malnutrition. Therefore, nutrition interventions and food aid programs tailored to the needs of HIV-infected pregnant women are of great importance, particularly those with low socioeconomic status and those with limited food intake during rainy seasons when food insecurity is at its peak. This analysis also presents a novel way to assess and analyze dietary intake patterns among pregnant women in sub-Saharan Africa.
